# Oxidative Stress Level as a Predictor of Anastomotic Leakage after Rectal Surgery

**DOI:** 10.1155/2021/9968642

**Published:** 2021-06-28

**Authors:** Jiajun Luo, Hongxue Wu, Yu Yang, Yue Jiang, Jingwen Yuan, Qiang Tong

**Affiliations:** Department of Gastrointestinal Surgery I Section, Renmin Hospital of Wuhan University, Wuhan 430060, China

## Abstract

**Background:**

Early diagnosis of anastomotic leakage (AL) after rectal surgery can reduce the adverse effects of AL, thereby reducing morbidity and mortality. Currently, there are no accepted indicators or effective scoring systems that can clearly identify patients at risk of anastomotic leakage.

**Methods:**

A prospective study with assessment of the diagnostic accuracy of oxidative stress level (CAT, SOD, MDA) in serum and drain fluid compared to white blood cell count (WBC), C-reactive protein (CRP), and neutrophil percentage (NEUT) in prediction of AL in patients undergoing elective rectal surgery with anastomosis.

**Results:**

Most of the oxidative stress indicators we detected are of considerable significance in the diagnosis of anastomotic leakage. The level of MDA on postoperative day (POD)3 (areas under the curve (AUC): 0.831) and POD5 (AUC: 0.837) in the serum and on POD3 (AUC: 0.845) in the drain fluid showed the same excellent diagnostic accuracy as the level of CRP on the POD3 (AUC: 0.847) and POD5 (AUC: 0.896).

**Conclusions:**

The overall level of oxidative stress in serum and drain fluid is a reliable indicator for the early diagnosis of anastomotic leakage after rectal surgery. More specifically, among the redox indicators analyzed, MDA has almost the same predictive value as CRP, which provides another useful biomarker for the early diagnosis of anastomotic leakage.

## 1. Introduction

Colorectal cancer (CRC) is the third most commonly diagnosed malignancy and the fourth leading cause of cancer-related deaths in the world [1]. It is widely accepted that colorectal cancer continues to be a severe problem. Colorectal surgery is long established as the mainstay treatment for colorectal cancer [2]. Rectal cancer accounts for about 67% of colorectal cancers and has a higher incidence of postoperative anastomotic leakage (AL) than colon surgery [3]. AL occurs in 4%-33% [4] of patients and is a major complication after restorative resection for rectal cancer that may adversely impact morbidity, mortality, and functional outcomes [5, 6]. Despite efforts to reduce AL occurrence, the incidence of AL has remained relatively unchanged over the last several years [7].

The ongoing occurrence of AL is due to many factors including those pertaining to surgical technique such as blood supply, tension, suture type, or device deployment, as well as, patient-related factors such as frailty, poor nutritional status, or chemoradiotherapy [8]. AL is clearly associated with the healing process following surgery of the gut. Reactive oxygen species (ROS) and oxidative stress have long been recognized as key components in wound healing [9]. Furthermore, data have shown that the production of specific ROS and the activation of specific formyl peptide receptors (FPRs) regulate intestinal wound healing [10]. Low concentrations of ROS production are necessary to ward off invading microorganisms and are crucial for cell survival signaling, but excessive ROS or impaired ROS detoxification causes oxidative damage, which may lead to AL [11].

At present, the surgeons' clinical risk assessment has a low predictive value for AL in rectal surgery [12]. Early diagnosis of AL is important to allow for alternative treatments to prevent morbidity and mortality. Many scholars have made great efforts to find early AL diagnostic markers. Previous studies have reported that C-reactive protein (CRP) [13–16], procalcitonin (PCT) [14], cytokines [17], lactate [18], and amylase [19] could help in the early diagnosis of AL, but limitations remain. Considering the important role of ROS in intestinal healing, we believe that the detection of ROS and oxidative stress levels could be a complementary method for the early diagnosis of AL. In this study, the levels of three redox indicators, catalase (CAT), malondialdehyde (MDA), and superoxide dismutase (SOD) will be determined in patients' serum and drain fluid and assessed for correlation with AL. We will also measure C-reactive protein (CRP) along with quantification of white blood cells (WBC) and neutrophil percentage (NEUT) for comparison to determine its diagnostic accuracy.

## 2. Materials and Methods

### 2.1. Patients

All patients were diagnosed with rectal cancer and underwent elective rectal surgery with primary anastomosis. The exclusion criteria included patients under 18 years of age, emergency operations, advanced cancers that were not amenable to curative resection, immunosuppression, and patients with severe infections or an American Society of Anesthesiologists (ASA) physical status ≥ 4. Preoperative workup included a physical examination, colonoscopy, computed tomography (CT), and chest X-ray or chest CT. The decision to perform laparoscopic or open surgery is based on the patient's condition and willingness. The diagnosis of AL is based on intestinal contents in the peritoneal drainage fluid and oral contrast agent exudation during fluoroscopy or CT examination. The present study was designed as a prospective observational pilot study. Written informed consent was obtained from all patients prior to enrolment. This study was approved by the Ethics Committee of Renmin Hospital of Wuhan University.

### 2.2. Analytical Methods

Blood samples were collected from patients on postoperative day (POD)3 and POD5. Blood was drawn by venipuncture, allowed to clot for 30 minutes, and then centrifuged (10 min, 1000×g). Serum was collected and kept frozen at –80° until examination. Drain fluid were collected on POD3 and POD5 and kept frozen at –80° until examination. The quantification of the WBC and NEUT was performed with a hematology analyzer. The C-reactive protein (CRP) concentration in serum was determined by the turbidimetric method with a CRP test. Catalase (CAT), malondialdehyde (MDA), and superoxide dismutase (SOD) levels in serum and drain fluid were determined using commercially available kits (Nanjing Jiancheng Bioengineering Institute, Nanjing, China).

### 2.3. Statistical Analysis

Statistical analyses were performed using SPSS Statistics 22.0 software. *P* values < 0.05 were considered statistically significant. The *χ*^2^ test for independence in a contingency table and Fisher's exact test were used. The diagnostic accuracy of tests was quantified using the area (AUC) under the receiver operating characteristic (ROC) curve.

## 3. Results

A total of 270 patients with rectal cancer undergoing elective rectal surgery were enrolled in this study. Of these, 14 patients underwent open rectal resection and 256 underwent laparoscopy surgery. Characteristics of patients are shown in Table 1. A total of 31 (11.5%) patients underwent neoadjuvant treatment. Sixteen (5.93%) patients developed AL. AL was diagnosed between postoperative days 4 and 10. During statistical analyses of AL vs. non-AL, no differences in demographic data except neoadjuvant treatment were observed.

**Figure 1:**
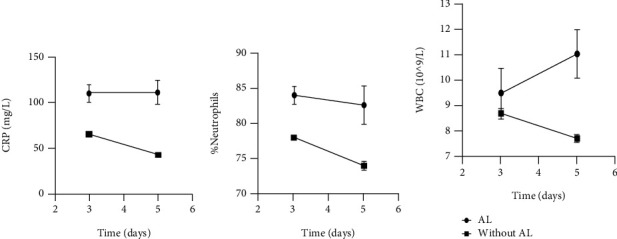
Mean levels of CRP (a), NEUT (b), and WBC (c) and relative error bars on POD3 and POD5.

Serum levels of CRP, WBC, and NEUT on POD3 and POD5 are illustrated in Table 2, and their changes are shown in Figure 1. The levels of all three serum factors were higher in the AL group than in the non-AL group and were statistically significant except WBC (POD3). The trends for CRP, WBC, and NEUT in the AL group were basically the same and increased overall with a slight decrease from POD3 to POD5, while all factors in the non-AL group were lower in comparison.

**Figure 2:**
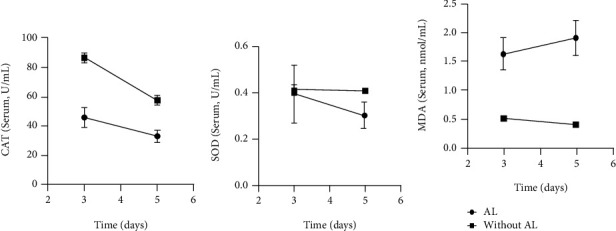
Mean levels of CAT (a), SOD (b), and MDA (c) and relative error bars on POD3 and POD5 in serum.

Serum levels of CAT, MDA, and SOD on POD3 and POD5 are illustrated in Table 3, and their changes are shown in Figure 2. The levels of CAT (POD3) and SOD (POD5) in the AL group were significantly lower than those in the non-AL group while the levels of MDA in the AL group were significantly higher than those in the non-AL group. Within the AL group between POD3 and POD5, CAT and SOD were decreased and MDA was increased, while in the non-AL group both CAT and MDA were decreased and SOD remained essentially unchanged.

**Figure 3:**
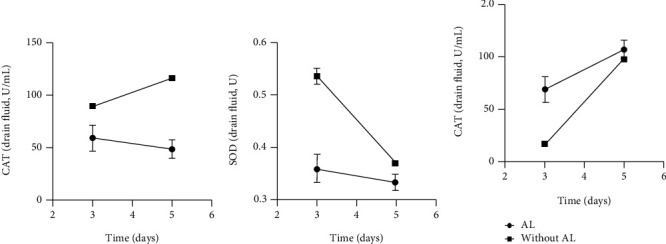
Mean levels of CAT (a), SOD (b), and MDA (c) and relative error bars on POD3 and POD5 in drain fluid.

Drain fluid levels of CAT, MDA, and SOD on POD3 and POD5 are illustrated in Table 4, and their changes are shown in Figure 3. The differences in their levels in drainage fluid are similar to those in serum and are statistically significant except for MDA (POD5). Within the AL group between POD3 and POD5, CAT and SOD were decreased and MDA was increased, while in the non-AL group both CAT and MDA were increased and SOD decreased.

**Figure 4:**
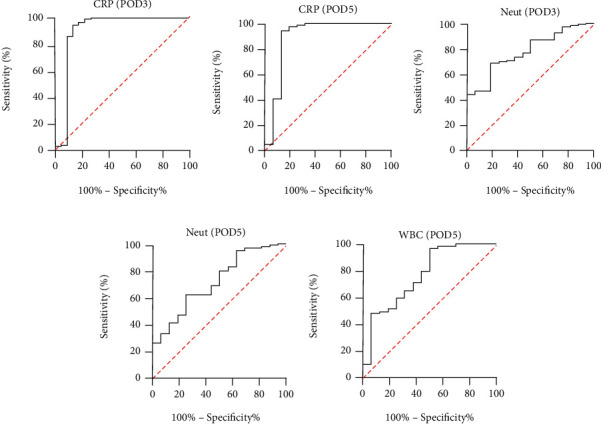
ROC curve analysis on POD3 for CRP (a) and Neut (c) and on POD5 for CRP (b), Neut (d), and WBC (e).

**Figure 5:**
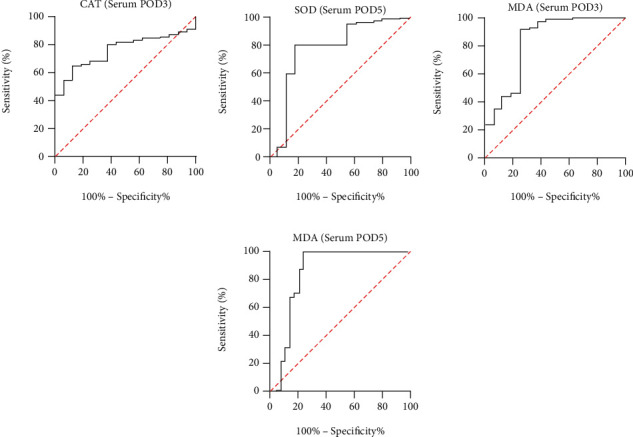
ROC curve analysis on POD3 in serum for CAT (a) and MDA (c) and on POD5 for SOD (b) and MDA (d).

**Figure 6:**
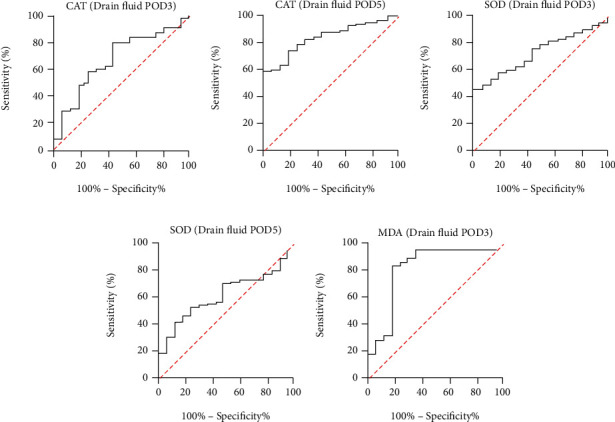
ROC curve analysis on POD3 in drain fluid for CAT (a), SOD (c), and MDA (e) and on POD5 for CAT (b) and SOD (d).

All the above indicators with statistically significant differences are drawn with ROC curves (Figures 4–6). The AUC ROC for CRP (POD3), NEUT (POD3), CRP (POD5), NEUT (POD5), and WBC (POD5) are 0.847, 0.779, 0.896, 0.719, and 0.766, respectively. The AUC ROC for CAT (POD3), MDA (POD3), SOD (POD5), and MDA (POD5) in serum are 0.759, 0.831, 0.769, and 0.837, respectively. The AUC ROC for CAT (POD3), MDA (POD3), SOD (POD3), CAT (POD5), and SOD (POD5) in drain fluid are 0.676, 0.845, 0.738, 0.836, and 0.635, respectively.

## 4. Discussion

For patients with rectal cancer, surgery is the main treatment method. The surgical method of rectal cancer has gradually developed from the initial partial resection to the expanded radical resection, and recently, it has been moving in the direction of precise and minimally invasive. The surgical method of rectal cancer is constantly being developed and improved. Laparoscopic rectal surgery has become the trend of modern colorectal surgery. Robotic rectal surgery has also been gradually implemented, and the concept of TaTME has also been proposed. With the deepening of the surgeon's understanding of perineum anatomy [20], the probability of complications of nerves and surrounding organs after rectal surgery is decreasing, but the incidence of anastomotic leakage does not seem to be significantly reduced. Compared with open surgery, the incidence of anastomotic leakage is similar in laparoscopic surgery and robotic surgery [21–23]. Early detection of anastomotic leakage is still an urgent problem.

At present, the most widely studied biomarker for prediction of AL is CRP [13, 14, 24–37]. NEUT and WBC counts are also candidate indicators; however, their value in prediction of AL is still controversial [38]. In our research, we first detected three indicators: CRP, NEUT, and WBC on POD3 and POD5, and CRP levels show a predictive effect as reported in the literature (AUC: CRP on POD3 = 0.847, CPR on POD5 = 0.896).

Whether in the serum or in the drain fluid, the oxidative stress indicators (CAT, MDA, and SOD) we have detected showed a diagnostic value for AL. The level of MDA exhibited the same superior diagnostic accuracy as the level of CRP (AUC: MDA (serum) on POD3 = 0.831, MDA (serum) on POD5 = 0.837, and MDA (drain fluid) on POD3 = 0.845).

It is well established that one of the main causes of AL is a decrease in anastomotic perfusion [39]. Studies have shown that tissue ischemia or necrosis often causes inflammation and oxidative stress, which further damages tissues [40]. The level of oxidative stress may reflect the degree of tissue ischemia. Another factor that is closely related to the healing of anastomosis is the deposition and metabolism of collagen [41]. Matrix metalloproteinases (MMPs) mediate collagen degradation, thereby increasing the risk of AL [42]. It is reported in the literature that oxidative stress injury can upregulate MMP expression, and the level of oxidative stress may reflect the level of MMP expression.

CAT, SOD, and MDA are commonly used oxidative stress indicators. In our study, we observed that compared with those of the non-AL leakage group, the levels of SOD and CAT in the serum and drain fluid of the AL group were significantly reduced, while the level of MDA was significantly increased. Our study found these indicators have a similar AL predictive effect as CRP. SOD and CAT are antioxidant enzymes, which constitute the primary cellular antioxidant defenses [43]. Studies have reported that the rise of SOD, CAT, and other antioxidant enzymes is an indication of tissue repair [44]. MDA has been used as a biomarker of oxidative stress. An increase of MDA reflects the enhancement of lipid peroxidation and tissue damage [45]. We hypothesize that the antioxidant system of patients with anastomosis is impaired, which leads to poor healing of anastomotic tissue.

## 5. Conclusions

In conclusion, the oxidative stress indicators we tested show great potential for the early diagnosis of AL in serum and drain fluid. Our research reveals that the level of MDA in serum or drain fluid has good diagnostic accuracy for AL. The oxidative stress level is expected to become a useful predictor of AL in the future.

## Figures and Tables

**Table 1:** Characteristics of rectal cancer (RC) patients.

Parameter	AL (*n* = 16)	Without AL (*n* = 254)	*P* value
Gender, M (%)	12 (75.0)	150 (59.1)	0.294
Age, mean ± s (years)	61.3 ± 10.3	62.7 ± 7.1	0.608
Comorbidities, *n* (%)			
Metabolic	3 (18.8)	13 (5.1)	0.060
Cardiovascular	6 (37.5)	52 (20.5)	0.120
ASA score, *n* (%)			0.544
1-2	12 (75.0)	172 (67.7)	
3	4 (25.0)	82 (32.3)	
Neo-adjuvant CRT, *n* (%)	5 (31.3)	26 (10.2)	0.010
Stage, TNM, *n* (%)			0.070
I	1 (6.2)	76 (29.9)	
II	7 (43.8)	54 (21.3)	
III	8 (50.0)	114 (44.9)	
IV	0 (0)	10 (3.9)	

**Table 2:** Comparison of CRP/WBC/NEUT on POD3 and POD5 in patients with and without anastomotic leak.

Parameter (mean ± SD)	AL (*n* = 16)	Without AL (*n* = 254)	*P* value
CPR_POD3_	109.8 ± 39.8	65.2 ± 22.3	<0.001
WBC_POD3_	9.492 ± 4.063	8.703 ± 3.086	0.332
NEUT_POD3_	84.02 ± 5.847	78.05 ± 5.368	<0.001
CPR_POD5_	111.2 ± 53.2	43.5 ± 18.4	<0.001
WBC_POD5_	11.054 ± 3.844	7.723 ± 2.477	<0.001
NEUT_POD5_	82.72 ± 10.96	74.01 ± 8.798	<0.001

CRP: C-reactive protein; NEUT: neutrophil percentage; WBC: white blood cells; POD: postoperative day.

**Table 3:** Comparison of CAT/MDA/SOD (serum) on POD3 and POD5 in patients with and without anastomotic leak.

Parameter (mean ± SD)	AL (*n* = 16)	Without AL (*n* = 254)	*P* value
CAT_POD3_	45.82±27.20	86.23±52.01	0.023
MDA_POD3_	1.629±1.119	0.531±0.309	<0.001
SOD_POD3_	0.394±0.503	0.409±0.394	0.885
CAT_POD5_	32.82±17.21	57.63±52.26	0.060
MDA_POD5_	1.912±1.211	0.419±0.233	<0.001
SOD_POD5_	0.302±0.218	0.408±0.127	0.002

CAT: catalase; SOD: superoxide dismutase; MDA: malondialdehyde; POD: postoperative day.

**Table 4:** Comparison of CAT/MDA/SOD (drain fluid) on POD3 and POD5 in patients with and without anastomotic leak.

Parameter (mean ± SD)	AL (*n* = 16)	Without AL (*n* = 254)	*P* value
CAT_POD3_	59.62 ± 48.50	90.06 ± 58.46	0.043
MDA_POD3_	0.906 ± 0.652	0.219 ± 0.205	<0.001
SOD_POD3_	0.358 ± 0.112	0.536 ± 0.237	0.003
CAT_POD5_	49.18 ± 35.68	116.5 ± 50.91	<0.001
MDA_POD5_	1.387 ± 0.556	1.294 ± 0.406	0.840
SOD_POD5_	0.332 ± 0.055	0.369 ± 0.096	0.014

CAT: catalase; SOD: superoxide dismutase; MDA: malondialdehyde; POD: postoperative day.

## Data Availability

The datasets used and analyzed during the current study are available from the corresponding author on reasonable request.
